# Alpha-cell glucagon is essential for maintaining β-cell function and identity in adult mice

**DOI:** 10.1016/j.jbc.2026.113145

**Published:** 2026-05-13

**Authors:** Krishna Prasadan, Mohamed Saleh, Madison Thomas, Omar Al Abyad, Chiyo Shiota, Justin Molitoris, Ting Zhang, Anuradha Sehrawat, Mohammad Tariq, Dhivyaa Rajasundaram, Sofia King, Ranjeet Kalsi, Xiangwei Xiao, Jon Piganelli, Farzad Esni, George K. Gittes

**Affiliations:** 1 Division of Pediatric Surgery, UPMC Children’s Hospital of Pittsburgh, Pittsburgh, Pennsylvania, USA; 2 Division of Pediatric Endocrinology, UPMC Children’s Hospital of Pittsburgh, Pittsburgh, Pennsylvania, USA; 3 University of Pittsburgh, Graduate School of Public Health, Human Genetics, Pittsburgh, Pennsylvania, USA

**Keywords:** glucagon gene, β-cell, glucose metabolism, insulin secretion, transgenic mice

## Abstract

Several studies have analyzed the effect of glucagon on β-cells and glucose homeostasis, either by ablating α-cells or by globally deleting the glucagon/glucagon receptor gene. To investigate possible interactions between α-cells and β-cells, and the effect of α-cells on β-cells/glucose homeostasis, we generated mouse models to allow deletion of the glucagon gene in α-cells (acute-α-GCG-KO) and the glucagon receptor gene in β-cells of adult mice. Specific deletion of the glucagon gene in the α-cell in adult mice led to impaired glucose tolerance, reduced β-cell mass and function, islet inflammation, β-cell endoplasmic reticulum stress, and altered β-cell ultrastructure. Notably, these detrimental effects were reversed by exogenous glucagon administration, but not by the glucagon-like peptide-1 analog exendin-4, indicating that glucagon deficiency specifically harms β-cells. Interestingly, acute ablation of α-cells in acute-α-GCG-KO mice reversed the alteration in glucose homeostasis and in β cells, suggesting that α-cells lacking glucagon gene expression can negatively impact β-cells, perhaps by some unknown factor(s). To investigate the role of the β-cell’s glucagon receptor on the observed effect of glucagon gene deletion, we specifically deleted the glucagon receptor gene in β-cells, either congenitally or acutely in the adult mouse; here, there were no changes in β-cells and glucose homeostasis, suggesting that the effect of glucagon on β-cells can be mediated *via* other signaling pathways. Conclusion: Glucagon gene deletion in α-cells of adult mice is detrimental to β-cells, and this effect is reversed by glucagon administration, suggesting that glucagon deficiency is specifically injurious to β-cells.

α- and β-cells are the two most prominent and numerous cells in the islets of Langerhans. Studies have shown that insulin secretion inhibits glucagon secretion (1). Conversely, glucagon has a stimulatory effect on β-cell function and insulin secretion (2). However, the overall paracrine effect of α-cell/glucagon on β-cells remains unclear. The role of α-cells/glucagon in the pathophysiology of diabetes has become more evident. In both impaired glucose tolerance and type 2 diabetes, insulin resistance at the hepatic level occurs due to both impaired insulin signaling (3, 4) and inappropriate hyperglucagonemia (5, 6), which results in increased hepatic glucose production and contributes to hyperglycemia in type 2 diabetes (7). Delineation of the paracrine interaction between α-cells and β-cells could help us to better understand the complex pathophysiology of type 2 diabetes. Several animal models have been established to study the paracrine effect of α-cells/glucagon on β-cells. Specific ablation of the α-cell transcription factor *Arx* led to a congenital loss of α-cells; these mice exhibited proliferation of β-cells with lower blood glucose and improved glucose tolerance (8). However, ablation of α-cells in neonatal (9) and adult (10) transgenic mice did not affect β-cell function or glucose homeostasis. Congenital deletion of glucagon and glucagon-related peptides caused α-cell hyperplasia in adult mice; also, these mice had normal glucose tolerance and lower insulin levels due to improved insulin sensitivity (11). In addition, other mouse models were generated to study the role of the glucagon receptor on β-cells and in glucose homeostasis; global deletion of the glucagon receptor caused α-cell hyperplasia and led to improved glucose tolerance in adult mice (12). Another study showed that islets isolated from glucagon receptor KO mice had impaired insulin secretion (13).

In the current study, we aimed to better understand the paracrine interaction between α-cells and β-cells by generating a mouse model that allows specific deletion of the glucagon gene in α-cells in adult mice (acute-α-GCG-KO) and demonstrated how it affects β-cells and glucose homeostasis compared to α-cell ablation. Moreover, we specifically deleted the glucagon receptor gene in β-cells in adult mice and studied the effect on glucose homeostasis as compared to previous studies focused on the global deletion of glucagon receptors.

## Results

### Deletion of the glucagon gene in α-cells results in loss ofβ-cell mass

**Figure 1:**
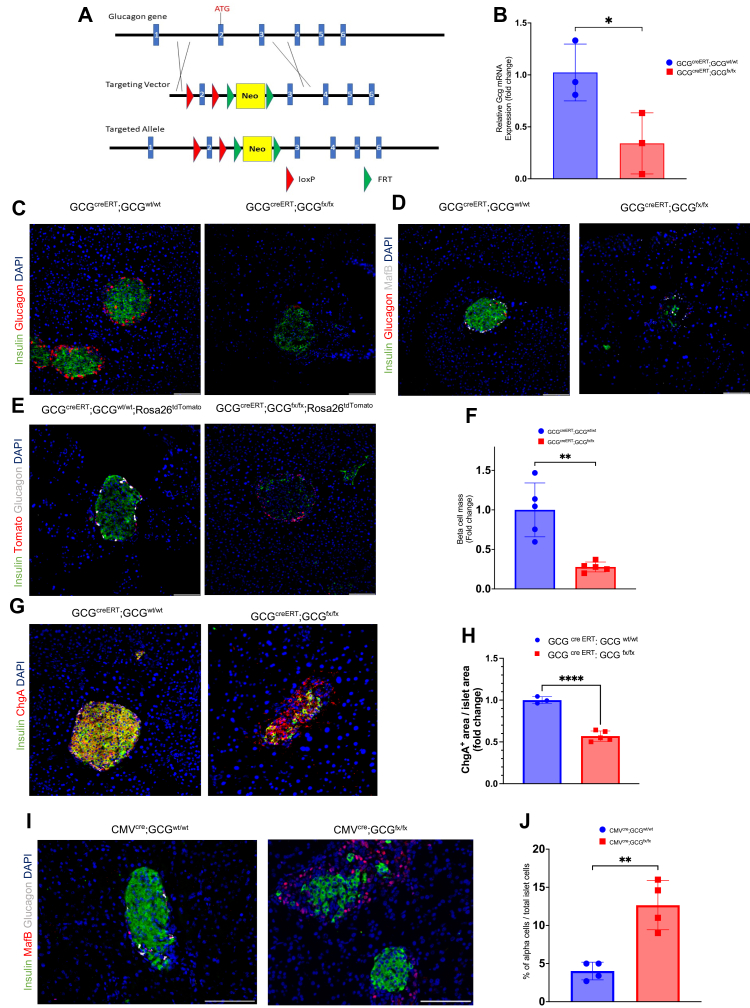
**Deletion of the glucagon gene in α-cells results in loss of β-cell mass.***A*, a schematic figure represents the *Gcg* gene locus, targeting vector, and targeted alleles. *B*, relative mRNA expression of Gcg normalized to Ppia, shown as fold change relative to GCG^creERT^;GCG^wt/wt^ mice (n = 3 in each group), *p* = 0.0425. *C* and *D*, immunostaining 8 weeks after glucagon deletion showed decreased insulin and glucagon staining in GCG^creERT^;GCG^fx/fx^ mice compared to littermate controls. *C*, similarly, there was decreased insulin and glucagon staining in GCG^creERT^;GCG^fx/fx^ mice compared to littermate controls. *D*, however, in GCG^creERT^;GCG^fx/fx^ showed *MafB*+; *glucagon-*cells, indicating deletion of glucagon in α-cells. *E*, lineage-tagged α-cells in GCG^creERT^;GCG^fx/fx^;Rosa26^tfTomato^ mice were *tomato*+/*glucagon*-, indicating deletion of glucagon in α-cells. *F*, β-cell mass quantification 8 weeks after glucagon deletion (n = 5/group). G and *H*, immunostaining for insulin and chromogranin A shows normal colocalization in littermate controls, while GCG^creERT^;GCG^fx/fx^ mice had significantly fewer *chromogranin A*+*/insulin* + cells. *I*, immunostaining for insulin and glucagon showed normal insulin staining with increased *glucagon* + cells in CMV^cre^;GCG^fx/fx^ mice compared to littermate controls. *J*, quantification of α-cells in CMV^cre^;GCG^fx/fx^ compared to littermate controls. Illustrative histology results from five animals are shown. The scale bar represents100 μm. The data are represented as the mean ± SD, ∗∗ = *p* < 0.01 and ∗∗∗∗ = *p* < 0.0001. Statistical analysis was performed by an unpaired two-tailed *t* test. CMV, cytomegalovirus; GCG, glucagon gene.

To study the effect of α-cell-derived glucagon on β-cell growth and function, we generated a mouse model that allows tamoxifen-dependent deletion of the glucagon gene in α-cells of adult mice. Here, we crossed glucagon GCG^fx/fx^ mice with GCG^creERT^ mice (Fig. 1*A*). At 14 weeks of age, IP tamoxifen was administered to activate the Cre recombinase enzyme. Eight weeks after the tamoxifen administration, there was no difference in body weight between GCG^creERT^;GCG^fx/fx^ mice (acute-α-GCG-KO) and their littermate controls (Fig. S1). A qPCR assay from pancreatic islets confirmed a significant reduction in *Gcg* mRNA in the acute-α-GCG-KO compared to their littermate controls (Fig. 1*B*). At that time, immunostaining of the pancreas showed near-total absence of glucagon, without α-cell hyperplasia (Fig. 1, *C* and *D*), as opposed to previous reports that showed α-cell hyperplasia with congenital deletion of glucagon (11). Here, we used *Maf-B* (Fig. 1*D*) immunostaining to identify α-cells in the absence of glucagon expression. To further confirm the lack of glucagon in α-GCG-KO mice, we used GCG^creERT^;GCG^fx/fx^;Rosa26^tdTomato^ mice; in these mice, tamoxifen injection led to lineage-tagging of α-cells with tomato red, while simultaneously acutely deleting glucagon. In this model, there was near total absence of glucagon with no apparent increase in the number of lineage-tagged α-cells following the tamoxifen-induced acute loss of glucagon (Fig. 1*E*). In addition, specific deletion of the glucagon gene in α-cells of adult mice resulted in loss of β-cell mass (Fig. 1, *C*–*F*) as well as degranulation of β cells, quantified using chromogranin A (Fig. 1, *G* and *H*). To further compare the congenital global loss of the glucagon gene *versus* the specific deletion of glucagon in adult mice, we made a congenital global glucagon KO using CMV^cre^ crossed into GCG^fx/fx^ mice. In line with previous reports, congenital global loss of glucagon using CMV^cre^;GCG^fx/fx^ mice led to α-cell hyperplasia, and there was no loss of insulin-positive cells (Fig. 1, *I* and *J*).

### Acute deletion of the glucagon gene in α-cells led to reduced insulin secretion and reduced expression of β-cell-specific transcription factors

**Figure 2:**
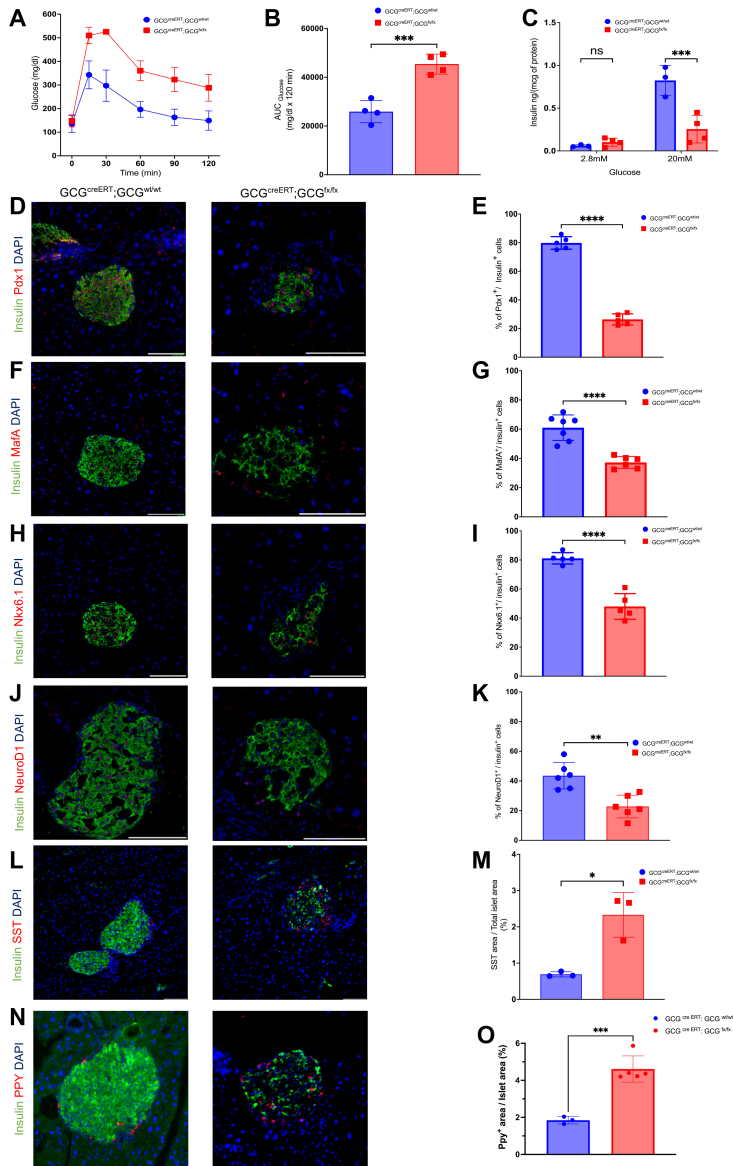
**Acute deletion of the glucagon gene in α-cells reduced insulin secretion and reduced the expression of β-cell-specific transcription factors.***A* and *B*, IPGTT 8 weeks after glucagon deletion comparing GCG^creERT^;GCG^fx/fx^ mice to littermate controls (n = 4/group) (*A*) area under the curve (AUC) analysis (*B*) showing a significant difference between the two groups. *C*, *ex vivo* GSIS comparing islets isolated from GCG^creERT^;GCG^fx/fx^ mice and their littermate control (n = 3–4/group). *D*, *E*, *F*, *G*, *H*, *I*, *J*, *K*, *L*, *M*, *N*, and *O*, representative images (*left panel*) with quantification (*right panel*) for coimmunostaining for insulin with *Pdx1* (*D* and *E*), *MafA* (*F* and *G*), *Nkx6.1* (*H* and *I*), *NeuroD1* (*J* and *K*), somatostatin (*L* and *M*), and pancreatic polypeptide (*N* and *O*). There are significantly fewer *MafA*+, *Pdx1*+, *Nkx6.1*+, and *NeuroD1*+ β-cells in GCG^creERT^;GCG^fx/fx^ mice compared to littermate controls, suggesting that the β-cells in GCG^creERT^;GCG^fx/fx^ mice are less mature than the β-cells of the littermate controls (*D*–*K*). In addition, there is a significant increase in the somatostatin+ and pancreatic polypeptide + area in the islet of the GCG^creERT^;GCG^fx/fx^ mice compared to the littermate controls, suggesting a shift in endocrine cell proportions in the GCG^creERT^;GCG^fx/fx^ mice (*L*–*O*). Illustrative histology results from five animals are shown. The scale bar represents 100 μm. The data are represented as the mean ± SD, ∗ = *p* < 0.05,∗∗ = *p* < 0.01 ∗∗∗ = *p* < 0.001, and ∗∗∗∗ = *p* < 0.0001. Statistical analysis for figure C was performed by two-way repeated-measures ANOVA followed by Holm-Šídák test for multiple comparisons and unpaired *t* test for *E*, *G*, *I*, *K*, *M*, and *O*. GCG, glucagon gene; GSIS, glucose-stimulated insulin secretion; IPGTT, intraperitoneal glucose tolerance testing.

At 14 weeks of age, IP tamoxifen was administered to GCG^creERT^;GCG^fx/fx^ mice and their littermate controls. Eight weeks after tamoxifen administration, intraperitoneal glucose tolerance testing (IPGTT) showed impaired glucose tolerance in adult acute-α-GCG-KO mice (Fig. 2, *A* and *B*) compared to their littermate controls. In contrast, congenital global loss of glucagon (CMV^Cre^;GCG^fx/fx^) did not alter glucose tolerance (Fig. S2, *A* and *B*). At the same time point, static glucose-stimulated insulin secretion (GSIS) analysis performed on islets isolated from acute-α-GCG-KO mice and their littermate controls showed impaired insulin secretion in acute-α-GCG-KO islets (Fig. 2*C*). These data suggest that acute deletion of the glucagon gene in mature α-cells leads to impaired β-cell insulin secretion and causes hyperglycemia. In addition, pancreata were harvested and stained for transcription factors important for normal β-cell function and maturation. Here, in the GCG^creERT^;GCG^fx/fx^ mice, we found a reduced expression of *pdx-1* (Fig. 2, *D* and *E*)), *maf-A* (Fig. 2, *F* and *G*), *nkx6.1* (Fig. 2, *H* and *I*), and *neuroD* (Fig. 2, *J* and *K*) compared to the littermate controls. To determine whether there is a concomitant change in other endocrine cell types in the acute-α-GCG-KO islets, we stained the acute-α-GCG-KO mouse islets for somatostatin, ghrelin, and pancreatic polypeptide and found a significant increase in the number of somatostatin-positive delta (δ) cells (Fig. 2, *L* and *M*), no change in ghrelin-positive cells (data not shown), and a significant increase in pancreatic polypeptide-positive cells (Fig. 2, *N* and *O*). To identify the source of increased δ cells in acute-α-GCG- KO mice, we used GCG^creERT^;GCG^fx/fx^;Rosa26^tdTomato^;Insulin1^dre^;Rosa26^GFP^ mice. Here, tamoxifen administration deletes glucagon and expresses tomato red lineage-tag in α-cells, while β-cells will be lineage-tagged with GFP. Somatostatin immunostaining in acute-α-GCG-KO mice after lineage tagging of both α-and β-cells showed GFP positive δ-cells, suggesting that there was conversion from β-cells into δ cells after acute glucagon loss (Fig. S3). In addition, pancreatic polypeptide immunostaining in the same mice after lineage tagging also showed GFP positive PP cells, once again suggesting conversion of β-cells into PP cells after acute glucagon loss.

### Acute deletion of the glucagon gene in α-cells alters β-cell ultrastructure and induces islet inflammation

**Figure 3:**
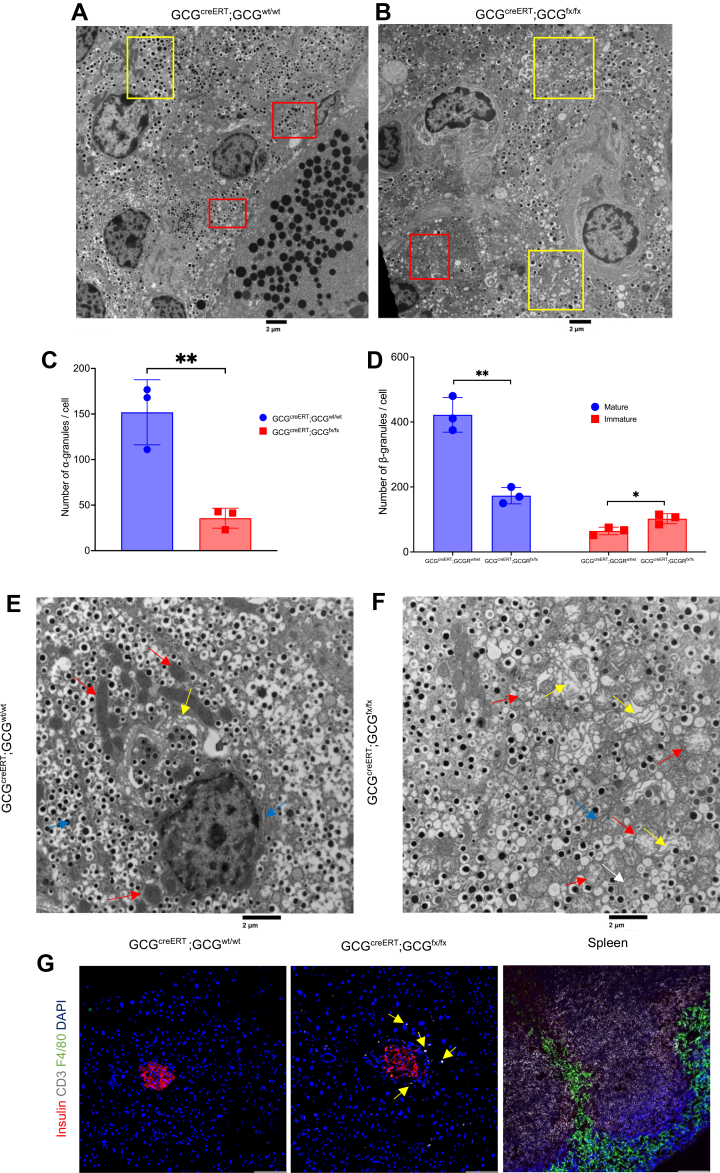
**Acute deletion of the glucagon gene in α-cells alters β-cell ultrastructure and induced islet inflammation.***A* and *B*, EM images of islets showing β-cells and α-cells in GCG^creERT^;GCG^fx/fx^ mice and their littermate controls, depicting decreased numbers of insulin granules on β-cells (*yellow area*) and decreased number of glucagon granules per α-cell (*red area*) in GCG^creERT^;GCG^fx/fx^ mice compared to the control group. *C* and *D*, quantification comparing the number of glucagon granules per α-cell (*C*) and number of insulin granules per β-cell (*D*) in GCG^creERT^;GCG^fx/fx^ mice compared to their littermate controls (n = 3/group), The scale bar represents 2 μm. *E* and *F*, ultrastructure of β-cells shows distorted and swollen mitochondria (*red arrow*), and dilated endoplasmic reticulum (*blue arrow*), and Golgi apparatus (*yellow arrow*) in the GCG^creERT^;GCG^fx/fx^ mice compared to controls. Also, phagocytic vacuoles (*white arrow*) were observed in the GCG^creERT^;GCG^fx/fx^ mice (*F*) but not in the control group (*E*). *G*, coimmunostaining mouse pancreata for insulin, CD3, and F4/80 showed increased CD3+ cells (*yellow arrows*) within and around the islets of GCG^creERT^;GCG^fx/fx^ mice compared to controls, while F4/80 staining for macrophages was negative in both groups. The spleen represents a positive control for CD3 and F4/80 immunostaining. Illustrative histology results from five animals are shown. The scale bar represents 100 μm. The data are represented as the mean ± SD, ∗ = *p* < 0.05,∗∗ = *p* < 0.01 ∗∗∗ = *p* < 0.001, and ∗∗∗∗ = *p* < 0.0001. Statistical analysis was performed by an unpaired *t* test. EM, electron microscopy; GCG, glucagon gene.

Eight weeks after tamoxifen administration, EM imaging of islets of acute-α-GCG-KO mice showed a significant reduction of α-granules in α-cells (Fig. 3, *A*–*C*). The EM images of α-cells are consistent with successful deletion of glucagon in this model. Interestingly, compared to normal littermate controls, the acute-α-GCG-KO β-cells had significantly decreased number of mature insulin granules with an increased number of the less dense immature insulin granules (Fig. 3, *A*, *B* and *D*). These data go in line with the decreased number insulin + cells, impaired β-cell function, and impaired glucose tolerance demonstrated in acute-α-GCG-KO mice (Figs. 1 and 2). In addition, in the acute-α-GCG-KO mice, β-cells had an abnormal ultrastructure, including rounded swollen mitochondria, significantly dilated endoplasmic reticulum (ER) cisternae, dilated Golgi apparatuses, and numerous phagocytic granules (Fig. 3, *E* and *F*). These changes are similar to the ultrastructural alterations seen in β-cells of mice fed a high-fat diet, and similar to what has previously been described in humans with type 2 diabetes (14, 15). These alterations suggest ER stress and mitochondrial dysfunction, which triggers oxidative stress that further worsens β-cell function and increases β-cell loss.

To further investigate the lack of glucagon on α-cells, bulk RNAseq was conducted on sorted α-cells from GCG^creERT^;GCG^fx/fx^;Rosa26^tdTomato^ or littermate controls. Eight weeks after tamoxifen administration, there was reduced expression of the glucagon gene (Gcg) in the GCG^creERT^;GCG^fx/fx^;Rosa26^tdTomato^ mice compared to littermate controls, confirming the KO (Fig. S4*A*). The GCG^creERT^;GCG^fx/fx^;Rosa26^tdTomato^ α-cells exhibited upregulation of Sox9 (16), a pancreatic progenitor marker, along with Tbx2 (16) and Nacc1 (17), suggesting loss of mature α-cell identity. In addition, genes associated with a progenitor or stem-like state were upregulated (Ngfr (18), Smo (19), Acvrl1 (20), Tead3 (21), Nacc1 (17), and Axin2 (22)), further suggesting loss of α-cell identity.

This was accompanied by a downregulation of mitochondrial function genes (Sdhb (23), Sdhd (23), Pemt (24), and Bcap31 (25)), lysosomal genes (Gm2a (26), Glmp (27), and Tspan31 (28)), antioxidant defense genes (Gstm5 (29), Gstt (29), and Prdx2 (30)), and ER homeostasis genes (Kdelr2 (31), Sec61G (32), and Trappc2 (33)). These results indicate mitochondrial dysfunction, impaired cellular stress response, and ER stress in the GCG^creERT^;GCG^fx/fx^;Rosa26^tdTomato^ α-cells.

Eight weeks after ablation of glucagon in acute-α-GCG-KO mice, pancreata showed an increase in lymphocytes within and around the islets (Fig. 3*G*). Also, there was no detectable signal for macrophages by immunostaining around or within the islet. To investigate the reason for the islet and peri-islet inflammation observed in this model, we performed a T-cell migration assay from islets isolated from acute-α-GCG-KO mice. Here, there was no T-cell migration from acute-α-GCG-KO islets, while islets isolated from NOD mice showed extensive T-cell migration (Fig. S5), suggesting that the increased T-cell population in the islets was an inflammatory response as opposed to an immune response. Further profiling of cytokines and chemokines in the islet milieu would help characterize the nature and source of this inflammation.

### Exogenous glucagon, as opposed to GLP-1, reverses glucose intolerance in acute-α-GCG-KO mice

In our model, the deletion of the glucagon gene will cause glucagon deficiency as well as deficiency in proglucagon-derived peptides, including glucagon-like peptide 1 (GLP-1) (34). To test whether β-cell loss and glucose intolerance in acute-α-GCG-KO mice are a direct consequence of loss of glucagon signaling *versus* GLP-1 signaling, we attempted to rescue hyperglycemia in acute-α-GCG-KO mice by administering exogenous glucagon or exendin-4 (GLP-1 analoge).

**Figure 4:**
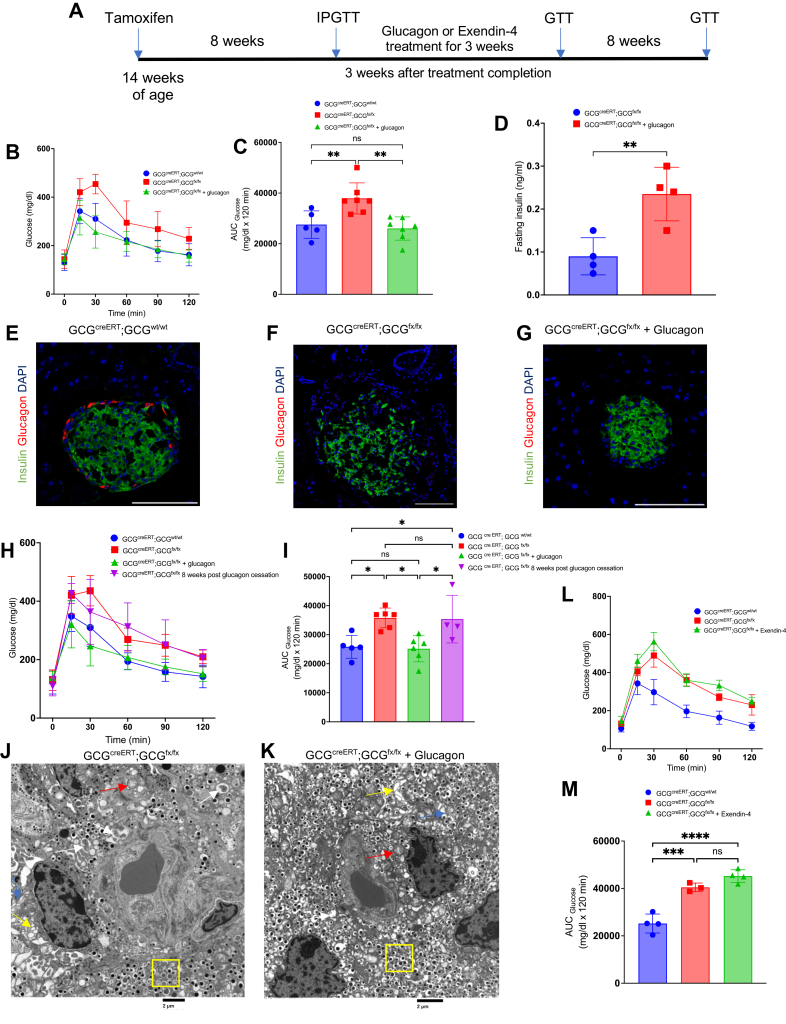
**Exogenous glucagon, not GLP-1 reversed glucose intolerance in acute-α-GCG-KO mice.***A*, schematic showing the experimental design and the schedule for glucagon and exendin-4 treatment. *B* and *C*, IPGTT 3 weeks after glucagon treatment comparing untreated GCG^creERT^;GCG^fx/fx^ mice, GCG^creERT^;GCG^fx/fx^ + exogenous glucagon treatment, and littermate controls (n = 5–7/group) (*B*). AUC analysis for the IPGTT (*C*). *D*, fasting plasma insulin levels comparing the GCG^creERT^;GCG^fx/fx^ mice GCG^creERT^;GCG^fx/fx^ + exogenous glucagon (n = 4/group). *E*, *F*, and *G*, immunostaining of islets for insulin and glucagon shows decreased insulin staining in the GCG^creERT^;GCG^fx/fx^ mice compared to littermate controls and GCG^creERT^;GCG^fx/fx^ + exogenous glucagon. *H* and *I*, IPGTT showed hyperglycemia GCG^creERT^;GCG^fx/fx^ mice compared to their littermate controls, reversal of the hyperglycemia after glucagon treatment in the GCG^creERT^;GCG^fx/fx^ mice, and recurrence of hyperglycemia in the GCG^creERT^;GCG^fx/fx^ 8 weeks after cessation of the glucagon treatment (n = 4–6/group). *J* and *K*, EM images of β-cells comparing GCG^creERT^;GCG^fx/fx^ mice to GCG^creERT^;GCG^fx/fx^ + exogenous glucagon, showing increased insulin granule (*yellow area*) in GCG^creERT^;GCG^fx/fx^ + exogenous glucagon mice compared to GCG^creERT^;GCG^fx/fx^, rounded swollen mitochondria (*red arrow*), dilated endoplasmic reticulum (*blue arrow*), and dilated Golgi apparatus (*yellow arrows*) in both groups, but less severe in the GCG^creERT^;GCG^fx/fx^ mice + exogenous glucagon. Also, the GCG^creERT^;GCG^fx/fx^ mice had increased phagocytic vacuoles (*white arrows*) compared to GCG^creERT^;GCG^fx/fx^ mice + exogenous glucagon. The scale bar represents 2 μm. *L* and *M*, IPGTT showed glucose intolerance in the GCG^creERT^;GCG^fx/fx^ and GCG^creERT^;GCG^fx/fx^ mice + exendin-4 compared to their littermate controls (*L*), AUC analysis for the IPGTT (*M*). Illustrative histology results from five animals are shown. The scale bar represents 100 μm. The data are represented as the mean ± SD, ∗ = *p* < 0.05,∗∗ = *p* < 0.01 ∗∗∗ = *p* < 0.001, and ∗∗∗∗ = *p* < 0.0001. Statistical analysis was performed by One-way ANOVA with repeated measures. AUC, area under the curve; GCG, glucagon gene; GLP-1, glucagon-like peptide-1; IPGTT, intraperitoneal glucose tolerance testing.

Eight weeks after acute glucagon deletion with tamoxifen, the acute-α-GCG-KO mice had impaired glucose tolerance (as shown in Fig. 2). Then, these mice received IP exogenous glucagon treatment twice a day for 3 weeks (Fig. 4*A*). Three weeks after completing glucagon treatment, there was a reversal of glucose intolerance in the acute-α-GCG-KO mice treated with glucagon (Fig. 4, *B* and *C*), and with increased fasting insulin levels compared to nontreated acute-α-GCG-KO mice (Fig. 4*D*). Immunohistochemistry showed that acute-α-GCG-KO mice treated with glucagon had increased insulin expression (Fig. 4, *E*–*G*) compared to untreated acute-α-GCG-KO mice. Eight weeks after discontinuation of the glucagon treatment, the IPGTT again showed impaired glucose tolerance in the acute-α-GCG-KO mice (Fig. 4, *H* and *I*). These data collectively suggest that glucagon deficiency specifically is injurious to β-cells in the adult pancreas.

Then, we analyzed the islet ultrastructure after glucagon treatment in acute-α-GCG-KO mice. Although not completely normalized, we found that glucagon treatment significantly improved the β-cell ultrastructural alterations that were observed in acute-α-GCG-KO (Fig. 4, *J* and *K*). However, the ultrastructural improvement was not back to normal, suggesting persistent or irreversible subcellular damage following prolonged glucagon deficiency.

Since GLP-1 receptors (GLP-1Rs) exist on β-cells, we wished to determine whether exogenous GLP-1 could rescue glucose intolerance in acute-α-GCG-KO mice. We thus repeated the experiment described above, except using exogenous exendin-4 treatment instead of glucagon (35, 36). We found that the exendin-4 did not reverse glucose intolerance (Fig. 4, *L* and *M*), suggesting that the lack of glucagon, and not GLP-1, was the cause of the islet ultrastructural alterations and glucose intolerance in acute-α-GCG-KO mice. We acknowledge that GLP-1R activity or endogenous GLP-1 levels were not directly measured in this study. However, the extremely short half-life of GLP-1 requires us to use exendin-4. The absence of phenotypic rescue by exendin-4 treatment strongly suggests that GLP-1 is not the primary mediator of β-cell dysfunction in this model.

**Figure 5:**
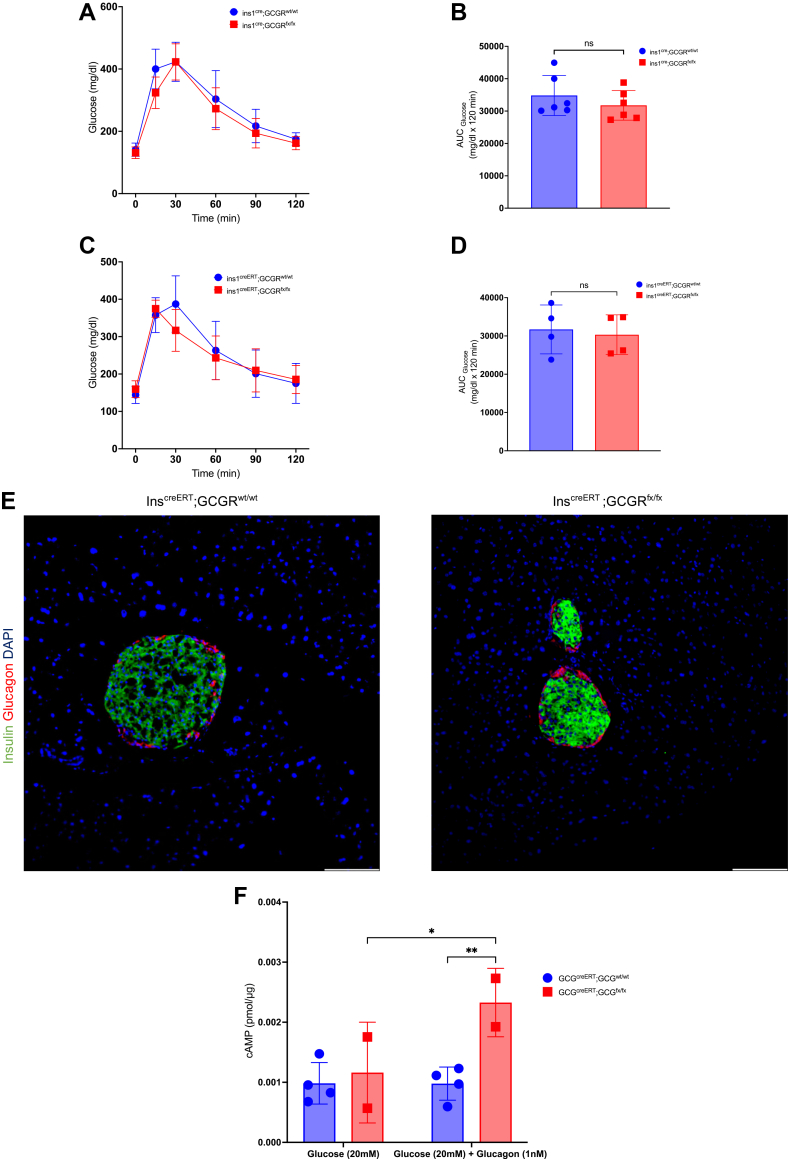
**Loss of glucagon receptors on β-cells of adult mice does not affect islet architecture nor glucose homeostasis.***A* and *B*, IPGTT at 8 weeks of age showed no significant difference in glucose tolerance between ins1^Cre^;GCGR^fx/fx^ and their littermate controls (n = 6/group) (*B*). AUC analysis for the IPGTT (*B*). *C* and *D*, IPGTT 16 weeks after deletion of GCGR showed no significant difference in glucose tolerance between ins1^CreERT^;GCGR^fx/fx^ and their littermate controls (n = 4/group) (*C*). AUC analysis for the IPGTT (*D*). *E*, immunostaining of islets for insulin and glucagon shows no apparent difference in insulin and glucagon staining between the ins1^CreERT^;GCGR^fx/fx^ mice and their littermate controls. Illustrative histology results from five animals are shown. *F*, cAMP assay from islets from the acute-α-GCG-KO and WT mice, exposed to either 20 mM glucose or 1 nM glucagon and 20 mM glucose. There was no difference in cAMP signaling between the acute-α-GCG-KO and littermate control mice when islets were exposed to 20 mM glucose. However, costimulating islets with 1 nM glucagon and 20 mM glucose significantly increased cAMP in the acute-α-GCG-KO islets compared to WT islets. The scale bar represents 100 μm. The data are represented as the mean ± SD, ∗ = *p* < 0.05,∗∗ = *p* < 0.01 ∗∗∗ = *p* < 0.001, and ∗∗∗∗ = *p* < 0.0001. Statistical analysis was performed by unpaired *t* test for figures B and D, Two-way ANOVA with multiple comparisons was used for figure F. AUC, area under the curve; GCGR, glucagon receptor; IPGTT, intraperitoneal glucose tolerance testing.

Loss of glucagon receptors on β-cells of adult mice does not affect islet architecture nor alter glucose homeostasis. To investigate the role of β-cell glucagon receptors (GCGRs) in the observed effect of glucagon on β-cells, we created a β-cell-specific GCGR KO mouse by crossing Ins1^cre^ mice with floxed GCGR mice (Ins1^cre^;GCGR^fx/fx^). In eight-week-old mice, specific deletion of glucagon receptors in β-cells did not alter glucose homeostasis (Fig. 5, *A* and *B*). Next, we generated a β-cell-specific conditional GCGR KO mice by crossing Ins1^creERT^mice instead of Ins1^cre^ mice with GCGR^fx/fx^ mice. Islets isolated from these mice 1 week after tamoxifen showed a significant reduction in GCGR mRNA (Fig. S6). , the acute deletion of the glucagon receptor gene in β-cells of adult mice did not alter glucose tolerance for up to 4 months following tamoxifen treatment (Fig. 5, *C* and *D*). Histology and immunohistochemistry of pancreata harvested 4 months after tamoxifen treatment showed normal islet architecture in the Ins1^creERT^;GCGR^fx/fx^ mice (Fig. 5*E*). These data are consistent with prior studies indicating that, while glucagon receptor signaling contributes to β-cell function, it is not essential for insulin secretion, in contrast to the more prominent role of GLP-1R signaling (37).

In addition, islets from the acute-α-GCG-KO and WT mice were exposed to 20 mM glucose and there was no significant difference in cAMP signaling between the two groups. These results suggest that hyperglycemia in the acute-α-GCG-KO mice is likely not related to the GCGR or its downstream cAMP signaling (Fig. 5*F*). However, costimulating islets with 1 nM glucagon and 20 mM glucose significantly increased cAMP in the acute-α-GCG-KO islets compared to WT islets, suggesting increased sensitivity to glucagon in β-cells from acute-α-GCG-KO mice (2).

These data collectively suggest that a β-cell-specific loss of the glucagon receptor in adult mice has no detectable effect on β-cell function and suggest that any effect of glucagon secretion from α-cells on β-cells is not primarily mediated *via* the glucagon receptor.

### Ablating α-cells after deletion of glucagon in acute-α-GCG-KO mice rescues β-cell function

**Figure 6:**
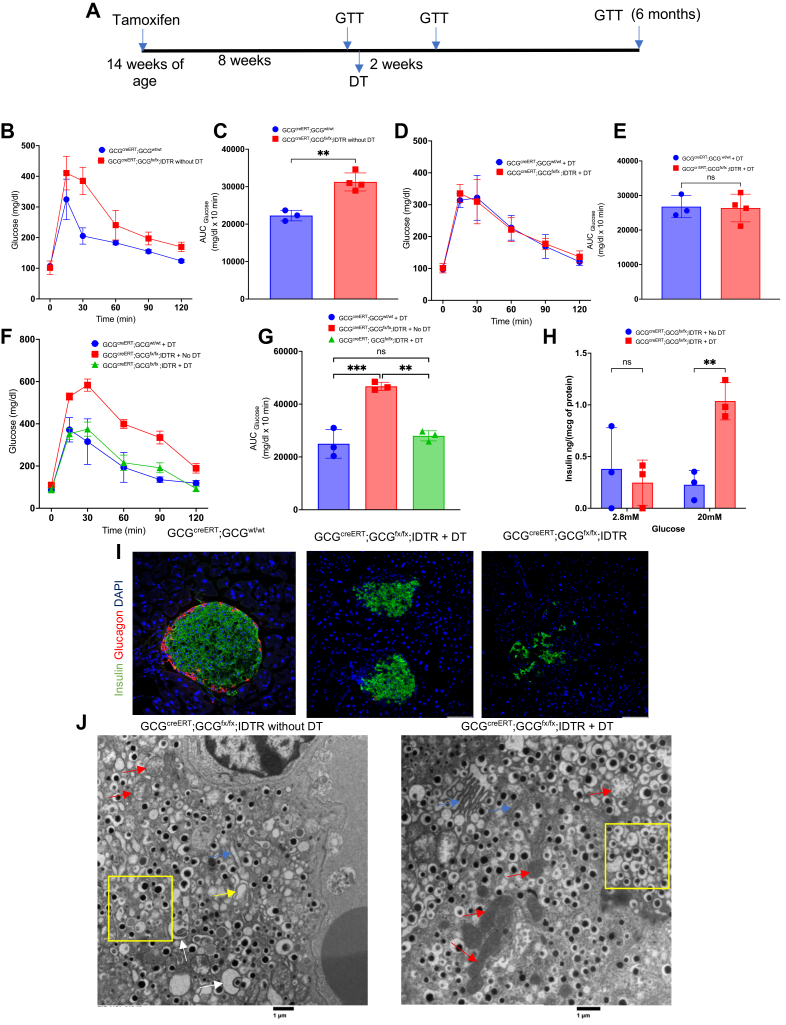
**Ablating α-cells after deletion of glucagon in acute-α-GCG-KO mice rescues β-cell function.***A*, schematic of the experimental design. *B* and *C*, IPGTT 8 weeks after glucagon deletion showed hyperglycemia in the GCG^creERT^;GCG^fx/fx^;Rosa^iDTR^ (without DT treatment) compared to their littermate controls (n = 3–4/group) (*B*). AUC analysis for the IPGTT (*C*). IPGTT 2 weeks after α-cell ablation showed no significant difference in the glucose tolerance between GCG^creERT^;GCG^fx/fx^;Rosa^iDTR^ + DT, and their littermate controls (n = 3–4/group) (*D*). AUC analysis for the IPGTT (*E*). *F* and *G*, IPGTT at 6 months after α-cell ablation showed hyperglycemia in the GCG^creERT^;GCG^fx/fx^;Rosa^iDTR^ mice without DT treatment, compared to their littermate controls; however, the α-cell ablation in the GCG^creERT^;GCG^fx/fx^;Rosa^iDTR^ + DT reversed hyperglycemia (*F*). AUC analysis for the IPGTT (*G*). *H*, Ex-vivo GSIS 2 weeks after α-cell ablation showed increased insulin secretion in islets isolated from the GCG^creERT^;GCG^fx/fx^;Rosa^iDTR^ + DT compared to islets isolated from GCG^creERT^;GCG^fx/fx^;Rosa^iDTR^ without DT treatment. *I*, immunostaining of islets for insulin and glucagon showed normal insulin and glucagon staining in the controls, normal insulin staining and nearly absent glucagon staining in the GCG^creERT^;GCG^fx/fx^;Rosa^iDTR^ + DT, however, GCG^creERT^;GCG^fx/fx^;Rosa^iDTR^ without DT treatment had decreased insulin staining and absent glucagon staining. *J*, EM images of β-cells comparing GCG^creERT^;GCG^fx/fx^;Rosa^iDTR^ + DT mice to GCG^creERT^;GCG^fx/fx^;Rosa^iDTR^ without DT, showing increased insulin granules (*yellow area*) in GCG^creERT^;GCG^fx/fx^;Rosa^iDTR^ + DT mice. There are rounded swollen mitochondria (*red arrows*), dilated endoplasmic reticulum (*blue arrows*), increased phagocytic vacuoles (*white arrows*), and dilated Golgi apparatus (*yellow arrows*) in GCG^creERT^;GCG^fx/fx^;Rosa^iDTR^ without DT mice; however, GCG^creERT^;GCG^fx/fx^;Rosa^iDTR^ + DT had improved β-cell ultrastructure, The scale bar represents 1 μm. Illustrative histology results from five animals are shown. The scale bar represents 100 μm. The data are represented as the mean ± SD, ∗ = *p* < 0.05,∗∗ = *p* < 0.01 ∗∗∗ = *p* < 0.001, and ∗∗∗∗ = *p* < 0.0001. Statistical analysis was performed *via* unpaired *t* test for figure *C* and *E*, One-way ANOVA for G and two-way repeated-measures ANOVA for figure *H*. Holm- Šídák test was used for multiple comparisons.AUC, area under the curve; GCG, glucagon gene; GSIS, glucose-stimulated insulin secretion; IPGTT, intraperitoneal glucose tolerance testing.

We and others have previously shown that ablation of α-cells in adult mouse does not alter glucose homeostasis (9, 10) as opposed to the specific acute deletion of glucagon in adult mice (Figure 1, Figure 2, Figure 3, Figure 4), where the presence of α-cells lacking glucagon appears to be detrimental to β-cell function. Thus, to test the possibility that the α-cells without glucagon were somehow detrimental to the β-cells, we conditionally ablated α-cells in acute-α-GCG-KO mice (Fig. 6*A*) by crossing the acute-α-GCG-KO mice with an inducible Diphtheria toxin receptor (Rosa26^iDTR^) mouse, so that tamoxifen treatment would simultaneously delete the glucagon gene and initiate expression of the DTR on α-cells. At 14 weeks of age, these Rosa26^iDTR^ acute-α-GCG-KO mice and iDTR-negative littermate controls received IP tamoxifen to delete glucagon and activate DTR expression in α-cells. As expected, 8 weeks post-glucagon deletion, IPGTT showed impaired glucose tolerance in the Rosa26^iDTR^ acute-α-GCG-KO mice (Fig. 6, *B* and *C*). Once glucose intolerance in the Rosa26^iDTR^ acute-α-GCG-KO mice was confirmed, the Rosa26^iDTR^ acute-α-GCG-KO mice and their DTR-negative littermate controls received diphtheria toxin to ablate α-cells. Two weeks after the ablation of α-cells, the IPGTT showed a complete reversal of hyperglycemia in the Rosa26^iDTR^ acute-GCG KO mice (Fig. 6, *D* and *E*). Also, an IPGTT performed 6 months post-DT continued to show similar normal glucose tolerance in ablated Rosa26^iDTR^ acute-α-GCG-KO mice compared to their DTR-negative littermate controls. (Fig. 6, *F* and *G*). In support of this *in vivo* data, *in vitro* GSIS of islets isolated from Rosa26^iDTR^ acute-α-GCG-KO mice 2 weeks after α-cell ablation showed increased insulin secretion compared to islets from DTR-negative littermate controls (Fig. 6*H*). In addition, immunohistochemistry of pancreata harvested from Rosa26^iDTR^ acute-α-GCG-KO mice 2 weeks after α-cell ablation showed a normal insulin immunostaining pattern (Fig. 6*I*). Also, the ultrastructure of β-cells in Rosa26^iDTR^ acute-α-GCG-KO mice after α-cell ablation showed a significant increase in the number of mature insulin granules (Fig. 6*J*). We also saw improvements in the structure of the mitochondria and ER, and a reduction in the number of phagocytic granules (Fig. 6*J*). These data together suggest that specifically, an acute glucagon KO in α-cells, and not the ablation of the whole α-cell is detrimental to β-cells. In other words, α-cells with deleted glucagon are detrimental to β-cell function.

## Discussion

In the present study, we have shown that specific deletion of the glucagon gene in α-cells of adult mice causes loss of β-cell mass, altered β-cell ultrastructure, impaired insulin secretion, and hyperglycemia. This detrimental effect on β-cells can be reversed by exogenous glucagon administration, but not by the GLP-1 analog exendin-4, suggesting that lack of α-cell glucagon, not GLP-1, is specifically injurious to β-cells. In addition, we have shown that deletion of the glucagon receptor on the β-cells of adult mice did not alter β-cell histology nor alter glucose homeostasis, suggesting that the glucagon receptor is not critical for any effect of glucagon on β-cells. Interestingly, we and others showed that in adult mice, the acute ablation of α-cells, as opposed to specific acute glucagon gene deletion in adult α-cells, did not alter β-cell morphology and function (9, 10), suggesting that α-cells lacking glucagon are somehow injurious to β-cells, perhaps due to another unknown α-cell factor. Although glucagon plays a critical role in counteracting hyperglycemia (38), some studies have found that glucagon is important for proper insulin secretion (37, 39, 40, 41, 42). Other studies have shown that glucagon is not essential for normal β-cell function. Our data strongly support an important role for glucagon in maintaining normal β-cell morphology and function, as shown in Figures 1 and 2. Furthermore, this finding was confirmed by observing the reversal of the altered islet histology and hyperglycemia, and improvement of β-cell ultrastructure in acute-α-GCG-KO mice after treatment with exogenous glucagon (Fig. 4).

As opposed to the current study, where we specifically deleted the glucagon gene in α-cells of adult mice, congenital global loss of glucagon and proglucagon-derived peptides did not alter glucose homeostasis, reduce insulin + cells, or result in α-cell hyperplasia (11). The differences here are likely related to adaptive changes that take place during pancreas development, including the α-cell hyperplasia that may overcome the alterations caused by glucagon gene deletion.

In line with the current study, adult mice with inducible ablation of α-cells recovered normal islet architecture and had normal glucose homeostasis (10). Here, the ablation of α-cells reversed the histological and functional alterations in β-cells in acute-α-GCG-KO mice. This finding suggests that α-cells lacking glucagon and glucagon-related peptides are somehow injurious to β-cells, possibly due to some unopposed negative factor. Determining this factor in the future may be of clinical significance to help understand the role of α-cells in diabetes and could be a potential therapeutic target.

In the acute-α-GCG-KO mouse model, we observed a significant increase in somatostatin-positive cells. Also, a previous study showed that global deletion of the glucagon receptor inhibited the progression of α-cells to maturity, affected the expression of several β-cell-specific genes, and resulted in an augmentation of the α-, β-, and δ-cell mass (43). We have shown that the increase in somatostatin-positive cells in the acute-α-GCG-KO mouse is likely due to β to δ differentiation (Fig. S3). Somatostatin-secreting δ-cells are stimulated directly by rising glucose levels, mediated by GLUT-1 and GLUT-3 receptors (44, 45). Also, previous studies demonstrated δ-cell hyperplasia in humans (46) and rodents (46, 47) with type 2 diabetes. These data suggest that the chronic exposure of δ-cells to hyperglycemia in acute-α-GCG-KO mice is the cause of the increase in somatostatin-positive cells.

In addition, we observed lineage-tagged β-cells that are pancreatic polypeptide-positive following ablation of the glucagon gene, suggesting differentiation of β-cells into PP-cells. Previous studies have shown that α-, β-, and δ cells can all be derived from lineage-traced PP-cells. Interestingly, β-cells derived from PP-cells are more resistant to hyperglycemia than non-PP-derived β-cells. Although GFP-positive somatostatin- and PP-positive cells suggest a possible conversion of β-cells into δ- and PP-cells, these findings require further validation through fate-mapping or transcriptomic analysis to confirm a true lineage transition *versus* transient marker expression.

The ultrastructure changes that took place after glucagon gene deletion in the α-cells (Fig. 4) mimic the changes noted in the high-fat diet mice including: decreased insulin granules, swollen mitochondria, and dilated ER. Also, deletion of the glucagon gene resulted in lymphocyte infiltration within and around the islets, but not macrophages, which suggests an inflammatory process rather than an immune response. Islet inflammation and infiltration with lymphocytes have been reported primarily in type 1 diabetes mouse models and patients with type 1 diabetes (48, 49, 50). Several studies have shown that the immune cell content of islets from rodents (51, 52) and humans (53, 54) with type 2 diabetes is mostly macrophages. In addition, and in line with the current study, previous reports showed that islets isolated from patients with type 2 diabetes had a similar pattern of islet and peri-islet infiltration with lymphocytes (55, 56), suggesting that the loss of glucagon and glucagon-related peptides results in β-cell ER stress similar to type 2 diabetes, which subsequently leads to islet inflammation. Several studies have shown that glucagon and GLP-1 have anti-inflammatory properties (57, 58, 59), which may explain the β-cell ER stress that develops in acute-α-GCG-KO mice. Also, the improvement in β-cell ultrastructure in the acute-α-GCG-KO mice after glucagon treatment, but not after GLP-1 treatment, suggests that glucagon is the key gene product for protecting β-cells from inflammation and ER stress. Again, these findings collectively suggest that α-cell alterations could have a role in the development of ER stress in β-cells and the progression of diabetes.

The effect of glucagon on β-cells is mediated through both the glucagon receptor and the GLP-1R (60, 61) by increasing the production of cAMP in β-cells (60). In the current study, specific deletion of the glucagon receptor on β-cells did not alter islet morphology and did not cause dysglycemia. A congenital global deletion of the glucagon receptor in mice resulted in hyperglucagonemia, improved glucose tolerance, α-cell hyperplasia, and δ-cell proliferation (12, 62). A similar phenotype to the global glucagon deletion was noted with specific deletion of the glucagon receptor on hepatocytes (63). Similarly, conditional global deletion of the glucagon receptor in the adult mouse resulted in improved glucose tolerance (64). These studies collectively suggest that the improved glucose tolerance associated with global deletion of the glucagon receptor is likely due to loss of the peripheral action of glucagon and improved insulin sensitivity (63), rather than being due to loss of any paracrine effect if glucagon on the β-cells. To that point, the specific deletion of glucagon receptor on β-cells shown in this study did not alter glucagon tolerance and did not affect islet morphology (Fig. 5). In line with our data, congenital deletion of glucagon receptor on β-cells did not alter glucose tolerance and did not affect *ex-vivo* glucagon stimulated insulin secretion (65). Conversely, congenital deletion of the GLP-1R resulted in impaired glucose tolerance and fasting hyperglycemia without detectable changed in fasting glucagon (66, 67). In addition, in mice with glucagon receptor KO, global GLP-1R deletion reversed the improvements in glucose tolerance without altering insulin sensitivity (67). Also, GLP-1R antagonist treatment reduced glucagon-stimulated insulin secretion by 65% in WT islets and 80% in glucagon receptor null islets (65), indicating that the GLP-1R is the primary mediator of glucagon-stimulated insulin secretion. These data collectively suggest that the glucagon receptor on β-cells is not critical for paracrine communication between α- and β-cells, as opposed to GLP-1R.

The limitation of our study is that acute deletion of the glucagon gene deletes glucagon and glucagon-related peptides, thus the subsequent effects of gene deletion may be due to glucagon hormone and/or glucagon-related peptide deficiency. We challenged this limitation by treating acute-α-GCG-KO mice with either glucagon or exendin-4; glucagon treatment resulted in improved glucose tolerance and improved β-cell ultrastructure, and this effect on glucose homeostasis is reversed 8 weeks after discontinuation of the glucagon replacement. On the other hand, treatment with the GLP-1 agonist had no effect and did not show improvements in glucose tolerance.

## Conclusion

Alpha cells and glucagon have important effects on β-cell structure and function, apparently mainly *via* glucagon signaling. The glucagon signaling to β-cells is primarily mediated by receptors other than the glucagon receptor, possibly the GLP-1R. In addition, α-cells lacking glucagon gene expression are somehow detrimental to β-cells, possibly *via* an unopposed unknown negative factor. In the future, it will be important to perform transcriptomic and proteomic analysis of the α-cell transcriptome to understand β-cell dedifferentiation and loss of identity with loss glucagon, and to pinpoint this potential negative factor. Further studies are needed to identify the signaling pathway of glucagon in β-cells and identify other α-cell factors that could play a role in the paracrine effect of α-cells on β-cells.

## Experimental procedures

### Animal models and treatments

All mouse experiments were approved by the Animal Research and Care Committee at the Children's Hospital of Pittsburgh and the University of Pittsburgh Institutional Animal Care and Use Committee.

• GCG^creERT^;GCG^fx/fx^(acute-α-GCG-KO): We have previously described a knock-in, tamoxifen-inducible Cre (creERT2) recombinase under the direction of the glucagon promoter (68) (Fig. 1*A*). The targeting vector for glucagon replaced the DNA sequence encoding the first 22 amino acids of preproglucagon in exon 2 with a creERT2 coding sequence, followed by an SV40 polyA (pA) signal (Fig. 1*A*). Immediately after the creERT2-pA sequence, we inserted a neomycin resistance gene, (neo) expression cassette flanked by flippase recognition target (Fig. 1*A*). The modified exon 2, along with 2 kb upstream and 7 kb downstream sequences, was retrieved into the PGKdtabpA plasmid exon two of the glucagon gene, followed by a neomycin cassette for selection. flippase recognition target sequences flanked the Neo cassette for subsequent removal from the vector using flippase recombinase. The exon-2 deletion produced a truncated N-terminal peptide and was never processed or secreted, ensuring the total deletion of glucagon family peptides.
• GCG^creERT^;GCG^fx/fx^;Rosa26^tdTomato^;Ins1^dre^;Rosa26^GFP^: We developed a novel Dre/RoxP system in combination with a Cre/LoxP system to create a dual lineage-tracking system in the developing and adult mouse pancreas (69). This mouse model will allow us to conditionally lineage-tag both α- and β-cells. First, we developed Ins1^dre^;Rosa26^GFP^ (Insulin1^dreHrpt^;Rosa26^GFP^) mice, then we crossed these mice with GCG^creERT^;GCG^fx/fx^;Rosa26^tdTomato^ mice. In this model, tamoxifen injection will delete glucagon gene expression and lineage-tag α-cells with tomato-red. Similarly, green fluorescence will label β-cells following insulin expression.
• CMV^cre^; GCG^fx/fx^: GCG^fx/fx^ mice were generated in our lab (9) and crossed with CMV^cre^ mice purchased from Jackson Labs (JAX stock#006054) (70).
• Conditional α-cell ablation model: IDTR mice purchased from Jackson Labs (JAX stock #007900) (71) were crossed with GCG^creERT^;GCG^fx/fx^ mice to express DTR in α-cells. Subsequent DT injections caused α-cell ablation.
• Ins1^cre^;GCGR^fx/fx^: We received the GCGR^fx/fx^ mice described previously (63) from Daniel J. Drucker, University of Toronto and were crossed with Insulin1^cre^ mice (JAX stock #026801) (72).
• In1s^creERT^;GCGR^fx/fx^: We received the GCGR^fx/fx^ mice described previously (63) from Daniel J. Drucker, University of Toronto and were crossed with Insulin1^creERT^ mice (JAX stock #026802) (72).

### Chemical treatments for mice

• Tamoxifen (Sigma-Aldrich): Mice received five daily doses of 75 μg/g IP.
• Diphtheria Toxin (List Biological Lab, Cat #150): mice received IP injections at dose of 50 μg/kg for three consecutive days.
• Glucagon: A dose of 5 μg glucagon (Boehringer Ingelheim, GTIN:00363323593032) was injected IP every 12 h for 3 weeks as described (73).

GLP-1: exendin-4 (Sigma-Aldrich, cat #141758-74-9) was given IP at dose of 1 nmol/kg once daily (74).

#### RT-qPCR

Islets were isolated as described below and cultured is rodent islet culture media overnight. Total RNA was extracted from mouse islets using the RNeasy Mini Kit (Qiagen, Cat #74104) according to the manufacturer’s instructions. RNA concentration and purity were assed with a nanophotometer (Implen N50-GO, Part #N50-GO). cDNA was synthesized with 150 ng of total RNA using the RevertAid First Strand cDNA Synthesis Kit (Thermo Fisher Scientific, Cat #K1622) with oligo (dT) priming according to the manufacturer’s protocol. qPCR was performed using TaqMan Fast Advanced Master Mix (Thermo Fisher Scientific, Cat #4444558) on a QuantStudio3 Real-Time PCR System (Thermo Fisher Scientific). Each reaction was run with a total volume of 10 μl containing 4 ng if cDNA. All samples were assed in triplicate. Cycling conditions followed the standard TaqMan Fast protocol: initial denaturation at 95 °C for 20 s, followed by 40 cycles of 95 °C for 1 s and 60 °C for 20 s.

Gene expression was quantified relative to the reference gene, *Ppia*, using the 2ˆ(−ΔΔCt) method. Data are expressed as fold change relative to the control group. Statistical analysis was performed on ΔCt values. Primer information.

• Glucagon (*Gcg*): Assay ID Mm00801714_m1
• Peptidylprolyl isomerase A (*Ppia*): Assay ID Mm02342430_g1
• Glucagon Receptor (*Gcgr*): Assay ID Mm00433546_m1

cAMP assay: Islets were isolated as described below and incubated at 37 °C overnight in rodent islet culture media. Following overnight culture, 30 islets per mouse washed in Kreb’s buffer. Next, all 30 islets were transferred to a new well containing 5 ml of 2.8 mM glucose solution and placed in the 37 °C incubator. After 1 h the 30 islets were divided into two groups of 15 and transferred to wells containing 5 ml of either 20 mM glucose or 20 mM glucose + 1 nM glucagon solutions. The islets were then placed in the 37 °C incubator for 30 min, finally the islets were collected in 200 μl of 0.1 M HCl for the cAMP assay. Samples obtained were stored immediately at −80 °C. cAMP ELISA kit (Enzo: ASB-OKEH03274) was then used to measure cAMP levels per the manufacturer’s instructions. The remaining samples were then used for protein quantification.

### *In vivo* glucose homeostasis studies

All the *in vivo* glucose homeostasis studies were performed on age- and sex-matched mice as described (75).

• IPGTT: Overnight 16-hour–fasted mice were injected IP with 2 g/kg glucose (Sigma-Aldrich). Blood glucose was measured from the tail vein at 0, 15, 30, 60, 90, and 120 min after injection using a glucometer (Contour NEXT EZ).
• ITT: Six-hour–fasted mice were received 0.75 U/kg of insulin (Humulin, Lilly) intraperitoneally, and blood glucose was measured at 0, 15, 30, 60, and 90 min after insulin injection.

### Islet isolation

Islets were isolated as described (76). Briefly, the pancreatic duct was infused and digested with type V collagenase (1.95 mg/ml). Islets were separated from the exocrine tissue with a discontinuous Ficoll gradient and then washed with Hanks’ Balanced Salt Solution (Gibco, Thermo Fisher Scientific) containing 20 mM Hepes buffer (Gibco, Thermo Fisher Scientific) and 0.2% bovine serum albumin (Sigma-Aldrich). Islets were then handpicked to eliminate any contamination from exocrine tissue.

### *Ex vivo* GSIS

*Ex-vivo* GSIS was performed as described (75, 76). Briefly, islets were harvested and placed in RPMI media overnight. Following overnight culture, 30 islets per mouse were preincubated in a 2.8 mM glucose solution for 30 min in a 37 °C incubator. Next, islets were washed in Kreb’s buffer twice and then transferred to a new well containing 2 ml fresh solution of 2.8 mM glucose. Immediately 100 μl of media was removed, labeled as time point 0, and stored. The islets were then incubated for 30 min in the 37 °C incubator, and subsequently, we removed 100 μl of media for time-point 1. Finally, the islets were transferred into a well containing 2 ml of 20 mM glucose solution for 30 min in the 37 °C incubator, and 100 μl of media was removed for time point 2. Samples obtained were stored immediately at −20 °C, and islets were then collected for protein quantification.

### RNAseq

Bulk RNAseq- The quality of the raw RNA-seq data was assessed using FastQC, poor quality ends were trimmed using Trimgalore (Felix Krueger, The Babraham Institute). High-quality sequences were aligned against the reference genome (Mm10) using STAR (77) aligner v2.7.11b, and counts were generated using featureCount (78). A list of differentially expressed genes was generated between the different conditions using DESeq2 (79) which applies a negative binomial model to account for overdispersion in count data. The resulting differentially expressed genes between the groups were defined at cut-off criteria of |log_2_ fold-change| ≥ 1.5 and *p*-value < 0.05 adjusted using the Benjamini and Hochberg’s approach for controlling the false discovery rate. To extract further biological insight from the samples, we implemented gene set enrichment analysis (80) which assesses the statistical enrichment of gene ontologies, and pathways, and visualized using Clusterprofiler v4.8.1 (81). All statistical analyses were performed using R 4.4.0.

### T-cell migration assay to isolate islet-infiltrating T-cells

A 96-well round-bottom plate was incubated overnight at 4 °C with complete media containing 0.5 μg/ml of CD3 and 1 μg/ml of CD28 antigens, and then washed with sterile PBS. Control and experimental islets (1–2 islets/well) were then cultured in the coated plate in 200 μl culture media (with Interleukin (20 U/ml) and IL-15 (2-5ηg/ml)) +3 percent pooled autologous mouse serum (to reduce nonspecific activation). Cultured islets were monitored for the outgrowth of infiltrating lymphocytes in a halo surrounding the islets every other day for 7 days.

### Tissue processing and histology

All pancreas samples were fixed with 4% paraformaldehyde for 24 h at 4 °C and then treated overnight with 30% sucrose at 4 °C. The tissues were then embedded in OCT, and 5 μm frozen sections were cut. For IHC, antigen retrieval was performed (heat and/or acid buffer). Slides were incubated with primary antibodies (Table S1) at 4 °C overnight. The following day the slides were incubated with fluorescent-conjugated (FITC, CY3, CY5) secondary antibodies (Jackson ImmunoResearch Labs) for 2 h at room temperature. Nuclear staining and mounting were performed using Fluoroshield with DAPI (Sigma-Aldrich).

### Transmission electron microscopy (TEM)

TEM was performed as described (76). Briefly, after fixation (2.5% glutaraldehyde, 2% paraformaldehyde in 0.1 M sodium phosphate buffer), tissues were rinsed twice in 0.1 M monosodium phosphate for 30 min and then placed in 1% osmium tetroxide in water for 1 h. Tissues were rinsed twice in deionized water. The samples were then dehydrated in serial concentrations of ethanol. Then tissues were placed were preinfiltrated in half resin/half propylene oxide overnight. The next day, tissues were infiltrated in 100% resin for 5 h, and were then embedded with fresh resin and polymerized at 60 °C overnight. The embedded tissues were sectioned with a Leica EM UC6 ultramicrotome. The sections were stained with 4% aqueous uranyl acetate for 30 min and 2 min in 0.2% lead citrate in 0.1 N sodium hydroxide. TEM imaging was performed using Thermo Fisher Scientific Ceta 16 MP camera. All TEM was performed by RK.

### Quantifications

The β-cell mass was quantified as described previously (76). Briefly, frozen sections at 50 μm intervals from the whole pancreas were immunostained for insulin and 4′,6-diamidino-2-phenylindole and imaged using a EVOS FL Auto two microscope. Captured images were analyzed using ImageJ software. Average β-cell mass was calculated by multiplying insulin + area/total pancreas area ratio by pancreatic weight. *Pdx1+*, *MafA+*, *Nkx6.1+*, and *NeuroD1+* β-cells were manually quantified from a minimum of six sections that were 100 μm apart for each mouse. At least 3000 cells were counted for each experimental condition. Counting continued beyond 3000 cells until 50 positive cells were tallied if the percentage of positive cells was low. To quantify δ cell area/total islet area, five pancreas frozen sections, 200 μm apart, were stained for insulin and somatostatin. Images were captured *via* Leica DMi8 inverted microscope and analyzed using ImageJ software. For hormone granules quantification in the EM images, total number of insulin and glucagon granules were quantified in a minimum of 10 equally sized cells obtained from three different sections/mouse.

### Statistics

AUC for GTT was calculated by the trapezoidal method. All data are displayed as mean and SD. Comparisons between two groups were made using unpaired and paired, 2-tailed *t* test as indicated. Comparison between multiple groups was made using o1- or 2-way ANOVA (with repeated measures where appropriate) followed by the Holm-Šidák test for multiple comparisons. *p* ≤ 0.05 was considered as statistically significant, and statistical tests were conducted using GraphPad Prism, version 8.3.

## Data availability

Most of the data described in the article are contained within the article. Any data not shown (immunostaining for ghrelin) is available upon request.

## Supporting information

This article contains supporting information.

## Conflict of interest

The authors declare that they have no conflicts of interest with the contents of this article.
