# Supplementary material for: Alpha-cell glucagon is essential for maintaining β-cell function and identity in adult mice
Source: J Biol Chem. 2026 May 13;302(7):113145. doi: 10.1016/j.jbc.2026.113145 (PMC13264239; doi:10.1016/j.jbc.2026.113145)
Supplement: Supporting Figures and Table [file mmc1.pdf]

Sup. Figure 1

A

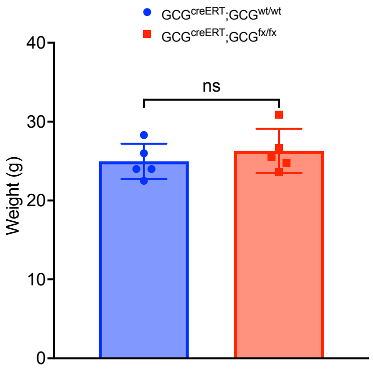

Body weight measurements 8 weeks after glucagon deletion showed no difference between GCG^{creERT};$GCG^{fx/fx}$ mice and their littermate controls (n=5/group). The data are represented as the mean ± SD. Statistical analysis was performed by unpaired t-test.

Sup. Figure 2

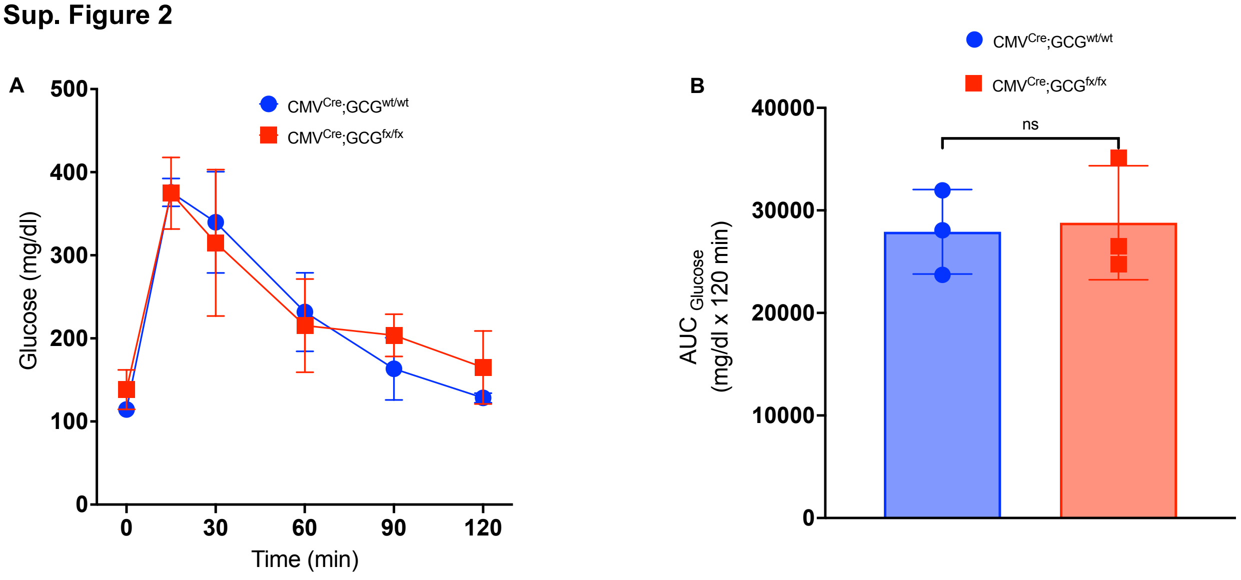

(A and B) IPGTT comparing CMV^{cre};GCG$^{fx/fx}$ mice and their littermate controls (n=3/group) **(A)**. AUC analysis for the IPGTT **(B)**. The data are represented as the mean ± SD. Statistical analysis was performed by unpaired t-test.

Sup. Figure 3

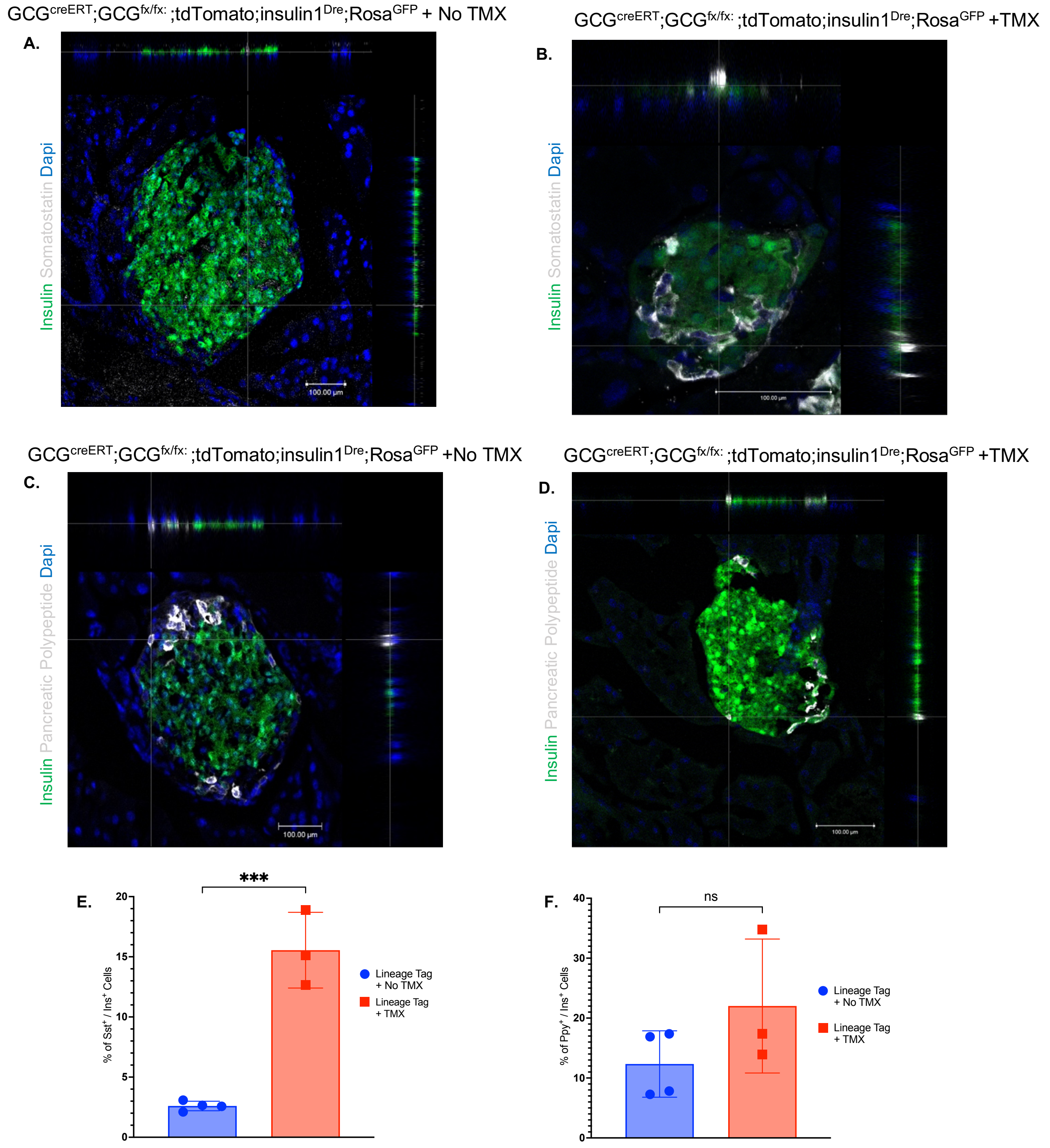

Immunostaining for somatostatin in control mice **(A)** compared to 8 weeks after glucagon deletion **(B)** in GCG^{creERT};GCG$^{fx/fx\ tdTomato}$;insulin1^{Dre};Rosa^{GFP} mice. Lineage-tagged β-cells are GFP^{+} somatostatin^{+}. Immunostaining for pancreatic polypeptide in control mice **(C)** compared to 8 weeks after glucagon deletion **(D)** in GCG^{creERT};GCG$^{fx/fx\ tdTomato}$;insulin1^{Dre};Rosa^{GFP} mice. Lineage-tagged β-cells are GFP^{+} pancreatic polypeptide^{+}. **(E)** Quantification of insulin positive and somatostatin positive area, p=0.0004. **(F)** Quantification of insulin positive and pancreatic polypeptide positive area, p=0.1862. Scale bars: 100 µm.

Sup. Figure 4

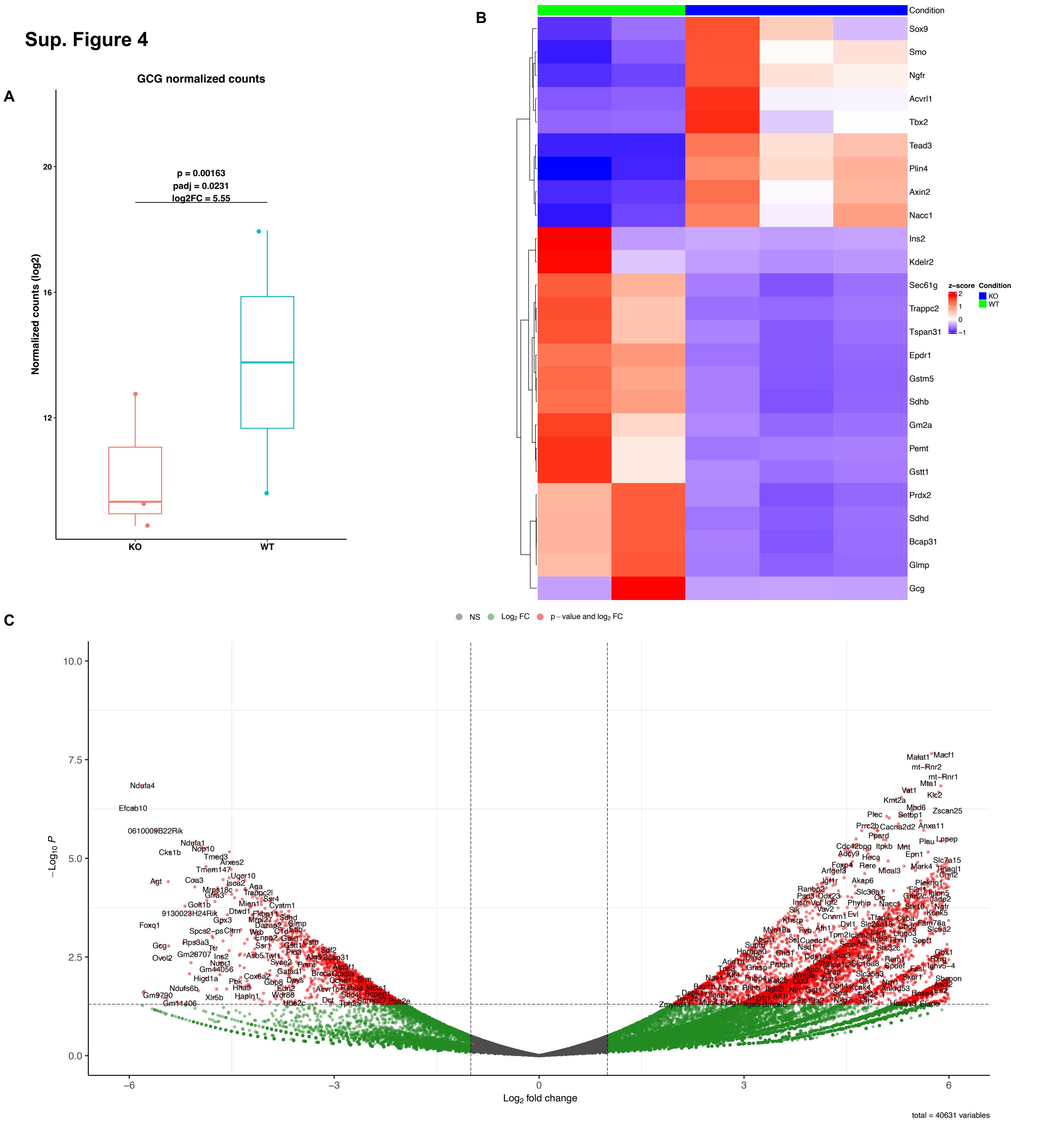

Bulk RNAseq from sorted α-cells from control mice (two leftmost green columns) and GCG^{creERT};GCG$^{fx/fx}$;Rosa26^{tdTomato} mice (three rightmost blue columns). **(A)** A violin plot showing differences in Gcg gene expression in the α-cells of GCG^{creERT};GCG$^{fx/fx}$;Rosa26^{tdTomato} mice compared to their littermate controls. **(B)** A heat map showing an upregulation of genes associated with loss of mature α-cell identity and a shift towards a progenitor-like state in the GCG^{creERT};GCG$^{fx/fx}$;Rosa26^{tdTomato} mice compared to controls. Genes associated with mitochondrial function, lysosomal function, antioxidant defense genes, and ER homeostasis are downregulated in GCG^{creERT};GCG$^{fx/fx}$;Rosa26^{tdTomato} mice compared to controls. **(C)** Volcano plot showing differential gene expression of the GCG^{creERT};GCG$^{fx/fx}$;Rosa26^{tdTomato} mice compared to littermate controls.

Sup. Figure 5

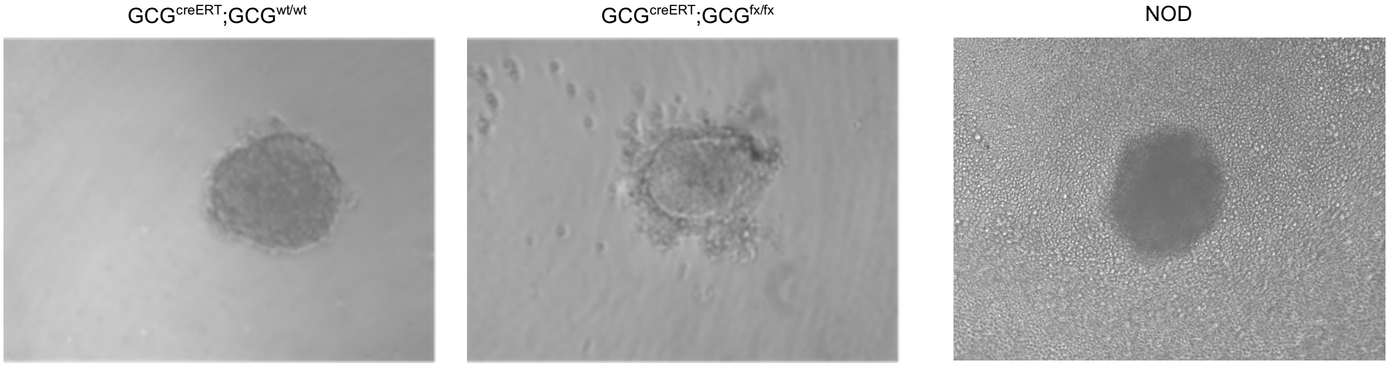

T-cell migration assay showing that islets in NOD mice had a substantial response whereas islets from GCG^{creERT};$GCG^{fx/fx}$ and their respective littermate controls did not show significant T-cell migration.

Sup. Figure 6

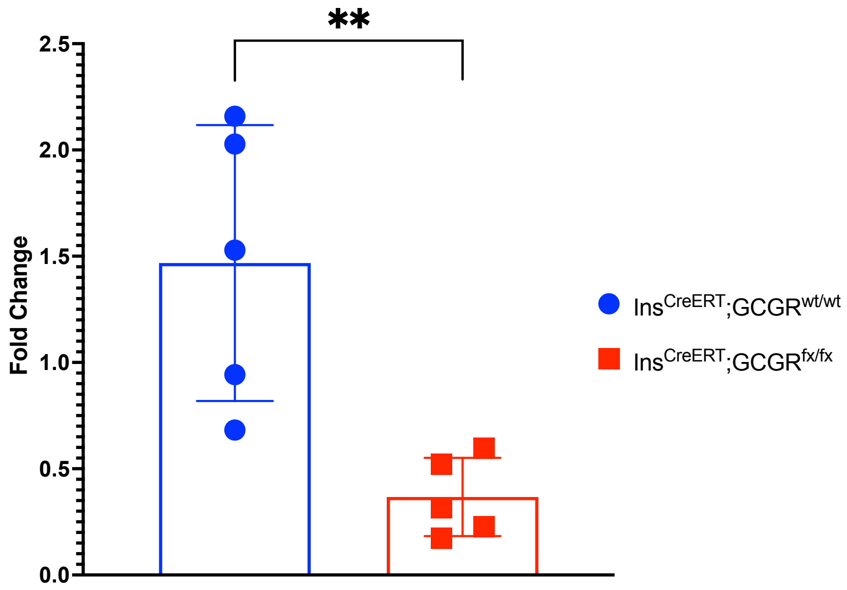

Ins^{CreERT};GCGR$^{fx/fx}$ and their Ins^{CreERT};GCGR$^{wt/wt}$ littermate controls were given tamoxifen at 8 weeks of age. One week after tamoxifen mice were sacrificed, their islets were isolated, RNA was extracted from islets, and converted into cDNA through reverse transcription. Quantitative PCR was done to determine GCGR mRNA expression in the islets. Ins^{CreERT};GCGR$^{fx/fx}$ mice had a significant reduction ($p=0.0065$) compared to littermate control mice.

Supplementary Table 1. Primary antibodies used in Immunohistochemistry staining

Name of Antibody	Manufacturer / Catalog Number	Source Species	Dilution Used
Insulin	Abcam/ab195956	Guinea Pig	1/500
Insulin	Abcam/ab181547	Rabbit	1/500
Glucagon	Abcam/ab92517	Rabbit	1/1000
Glucagon	Abcam/ab10988	Mouse	1/1000
Pdx1	Abcam/ab47308	Guinea Pig	1/100
MafA	Santa Cruz Biotechnology/sc-390491	Mouse	1/100
Nkx6.1	Abcam/ab221549	Rabbit	1/200
NeuroD1	Abcam/ab213725	Rabbit	1/100
Somatostatin	Abcam/ab111912	Rabbit	1/200
CD3	Dako/A0452	Rabbit	1/100
F4/80	Abcam/ab6640	Rat	1/300
Chromogranin A	Abcam/ab272732	Rabbit	1/500
Pancreatic Polypeptide	Abcam/ab283265	Rabbit	1/500
